# Music influences performance without increasing perceived exertion during high-intensity rowing intervals: a cross-over design study

**DOI:** 10.3389/fpsyg.2024.1427373

**Published:** 2024-08-16

**Authors:** Andrea Schittenhelm, Tom Brandt, Denny Andres, Patrick Adler, Merle T. Fairhurst, Annette Schmidt

**Affiliations:** ^1^NextGenerationEU, dtec.bw Project Smart Health Lab, Faculty of Human Sciences, Institute for Sports Science, Chair of Sport Biology, University of the Bundeswehr Munich, Neubiberg, Germany; ^2^Institute for Sports Science, University of the Bundeswehr Munich, Neubiberg, Germany; ^3^Centre for Tactile Internet with Human-in-the-Loop (CeTI), 6G Life, Faculty of Electrical and Computer Engineering, Technische Universität Dresden, Dresden, Germany; ^4^Research Center Smart Digital Health, Faculty of Human Sciences, Institute for Sports Science, Chair of Sport Biology, University of the Bundeswehr Munich, Neubiberg, Germany

**Keywords:** self-selected music, heart rate, recovery, pacing, drop in performance

## Abstract

**Objectives:**

This study assessed how exposure to slow (SBM) versus fast beat music (FBM) during high-intensity rowing intervals affects performance, heart rate (HR), lactate levels, relative perceived exertion (RPE), and recovery.

**Methods:**

The A/B crossover design involved 21 participants performing 5 × 500 m rowing intervals under two conditions: FBM and SBM. Primary endpoint was the difference in total rowing time. Secondary endpoints included average HR, average RPE as well as rowing interval times, RPE, and HR per interval. For exploratory purpose, HR and lactate drop during the initial 5 min post completion was analyzed.

**Results:**

Listening to FBM resulted in significantly shorter total rowing times (*p* = 0.009, r_B_ = 0.59), especially during the 1st interval. The 1st interval was also significantly faster than intervals 2–5 (*p* < 0.001), with the greatest performance drop between the 1st and 2nd interval during FBM. Average HR was significantly lower when listening to SBM (*p* = 0.03, r_B_ = 0.48), while average RPE showed no significant difference (*p* = 0.47, r_B_ = 0.02). Lactate values after interval 5 were significantly lower in SBM (*p* = 0.05, r_B_ = 0.41), but no significant difference was found for lactate drop (*p* = 0.21, r_B_ = 0.21). However, participants exhibited a higher HR drop rate with SBM (*p* = 0.05, r_B_ = 0.42).

**Conclusion:**

FBM improved performance without increasing RPE, while SBM proved superior for recovery. Systematic customization of music based on intended training stimuli holds broad potential for the competitive sports, fitness, and health sector.

## 1 Introduction

Music is a universal cultural phenomenon that elicits a multitude of physiological and psychological responses. Previous research has concluded that the underlying mechanisms are related to the modulation of brain activity of cortical systems involved in the hypothalamic–pituitary–adrenal axis, autonomic regulation, movement control, emotional-behavioral control, attention, and automatic evaluation (Koelsch, 2014). As a result, simply listening to music can lead to changes in respiration, heart rate (HR), heart rate variability, and blood pressure. Moreover, music has been found to improve physical performance, mask fatigue, increase arousal, regulate emotions, and benefit physiological efficiency in physical activities (North et al., 2000; Greasley and Lamont, 2006; Simpson and Karageorghis, 2006; Saarikallio and Erkkilä, 2007; Mohammadzadeh et al., 2008; Karageorghis et al., 2009; Saarikallio, 2011; Karageorghis and Priest, 2012a,b; Moore, 2013; Uhlig et al., 2013; Mclaughlin et al., 2020). Consequently, the influence of music could offer considerable potential in the field of sports to enhance athletes’ performance. The scientific literature provides emerging evidence towards this notion, whereby several parameters such as tempo, personal preferences, time of music exposure as well as the exercise intensity appear to influence the effect music has during exercise.

For instance, Crust showed that listening to music positively affects isometric muscular endurance. However, this effect only occurred if the music was listened throughout the exercise whereas being exposed to music solely before exercise did not (Crust, 2004). Positive effects on peak and mean power when listening to music during exercise compared to no music were also found by Stork et al. for sprint interval sessions on a cycling ergometer (4 sets of 30 seconds [sec] at maximum effort) whereas no differences between conditions were found for relative perceived exertion (RPE) (Stork et al., 2015). Improved performance was confirmed by Rendi et al. who tested the influence of fast beat (FBM), slow beat (SBM), and no music during 500 meter [m] rowing sprints. In this case, it was noteworthy that despite a shorter time to completion when listening to FBM, RPE did not differ compared to the other conditions. When comparing the results of Rendi et al. to other studies, it needs to be considered that the music was selected by the researchers and the participants were experienced rowers (Rendi et al., 2008). Similarly, Wu et al. showed that the time to perceived fatigue extended when listening to SBM and FBM compared to no music (Wu et al., 2022). Besides tempo and time of exposition, personal music preference seems to be another important factor (Ballmann et al., 2021). In this regard, Nixon et al. analyzed how listening to preferred compared to non-preferred music volume affects time to completion, HR, RPE, and motivation during a 2000 m rowing time trial. In contrast to the above-stated study of Rendi et al., time to completion did not differ between conditions. Nevertheless, RPE was significantly lower and motivation higher when listening to preferred music volume (Rendi et al., 2008; Nixon et al., 2022). Karow et al. also compared the effects of preferred, non-preferred, and no music in a 2000 m rowing time trial. Although the music was only played during the warm-up prior the actual time trial, relative power output and HR were higher while trial time was lower in the preferred music condition when compared to the other conditions whereas no differences occurred for RPE (Karow et al., 2020). Karageorghis and Priest concluded in a review that music could reduce RPE by ~10% during physical tasks. However, the authors stated that this effect mainly occurs at low to moderate intensities but is not pronounced in activities beyond the anaerobic threshold, respectively, high-intensity training (Karageorghis and Priest, 2012a,b). It was further hypothesized that external sensory stimuli could not be processed effectively during high-intensity exercise as the overwhelming physiological stimuli already overtax the capacity of the afferent nervous system (Rejeski, 1985; Hutchinson and Tenenbaum, 2007; Karageorghis and Priest, 2012a,b). In a systematic review of Marques et al. analyzing specifically the influence of music on high-intensity exercise, it was concluded that music does not reduce RPE (Marques et al., 2024).

To summarize, despite emerging evidence regarding the positive effects of music applied during exercise, scientific literature is still inconclusive and partly contradictory, especially in terms of high-intensity activities. However, based on the findings of Marques et al., the chances of benefiting from positive effects of music during high-intensity exercise could be increased by applying high tempo, preferred, motivational music and play it over the course of several high-intensity bouts (Marques et al., 2024). A form of exercise that is performed at high intensities in different fitness domains is rowing (Glassman, 2020; Bergmann, 2024). However, previous research investigating the influence of music during rowing did not adhere to the guidelines recently stated by Marques et al. (2024). Subsequently, the aim of the present study was to investigate how exposition to SBM compared to FBM which is self-selected and played during high-intensity rowing interval protocol affects performance, physiological parameters, RPE, and recovery.

## 2 Materials and methods

### 2.1 Experimental design

**Figure 1:**
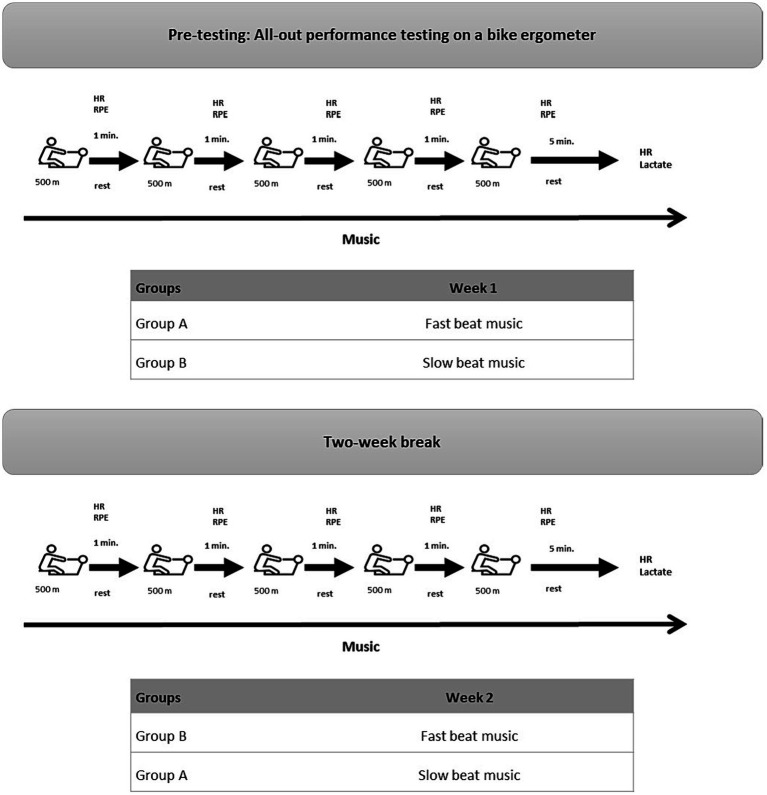
Study design. HR, heart rate; min, minutes; m, meters; RPE, relative perceived exertion.

This study followed an A/B crossover design in which the participants performed a 5 × 500 m rowing ergometer test under different music conditions (FBM and SBM) that varied in music tempo (beats per minute [bpm]). Before the actual test, a pre-test was carried out on a bicycle ergometer to ensure an even group allocation of participants according to their physical fitness. To achieve a power of 80% on a one-sided 5% significance level in the primary endpoint, a sample size of 22 was calculated by *a priori* power analysis with G*Power (Faul et al., 2007). The study protocol is displayed in Figure 1.

### 2.2 Procedure

#### 2.2.1 Pre-test

Before the testing on the rowing ergometer, a pre-testing was conducted at the University of the Bundeswehr Munich (UniBw M). During the pre-testing, demographic data and preferred music style were assessed. Additionally, an all-out test on an Excalibur sport bike ergometer (Lode BV, Groningen, Netherlands) was conducted to allocate participants evenly to groups A and B based on their physical performance abilities. The test protocol started at 70 watts [W] and was increased by 30 W every 2 min [min] until a maximum of 400 W. The participants were informed to cycle permanently at 60 to 80 turns/min. Otherwise, the testing was terminated within 10 sec after dropping below the threshold. Shortly before switching to the next higher stage, HR, blood lactate, and RPE were recorded. After quitting due to maximum exertion or reaching 400 W, the participants cycled another 5 min at 50 W for recovery.

#### 2.2.2 Music characteristics

In the literature focusing on music in physical activity, music with <120 bpm is considered slow and > 120 bpm fast (Terry et al., 2020). This was taken into account for the present study to categorize the music conditions. Two music conditions were chosen: (A) FBM (mean (M) = 134.2; standard deviation (SD) = 33.2 bpm) and (B) SBM (*M* = 96.9; SD = 16.8 bpm). Based on the given mean tempo of each track in bpm, the mean tempo of the SBM and FBM condition was calculated. The FBM condition was divided into 2 music sets according to the participant’s preferences. In addition to music tempo, music tracks were selected based on the following principles: (A) the FBM tracks had a cheerful music style and should induce positive emotions, (B) the SBM tracks had no lyrics and were marketed as “Yoga” or “relaxing” music by the streaming provider. The music was played by use of a smartphone and Bluetooth speakers at a volume of 60–65 decibel [dB]. Participants were instructed to not listen to any music for at least 1 hour [h] before the test. The music was started when the participants began their warm-up and then played throughout the entire protocol, including rest periods and cool-down.

#### 2.2.3 5 × 500 m test

The actual testing took place at the military affiliation CrossFit Kokoro^®^ and consisted of 2 test sessions with a 2-week break in between. Participants were instructed to stop eating and to not listen to any music at least 1 h before the sessions. In the 1st test session, group A rowed while listening to FBM and group B to SBM. During the 2nd session, music conditions were interchanged. Both sessions followed the same rowing protocol. As recommended for high-intensity exercise testing, a rowing interval protocol was chosen that allowed participants to reach their limit of tolerance in about 10 min (Porszasz et al., 2003). According to previous research including physically active adults (*N* = 17, Age: 29 (SD = 5) years), 500 m rowing on an ergometer takes 112.1 (SD = 7.9) and 113.2 (SD = 8.9) sec being in a pre-exhausted state (Silva de Souza et al., 2024). Therefore, 5 × 500 m intervals were determined as a test that physically active sports science students would be able to carry out.

The protocol started with a guided warm-up. Thereafter, participants were instructed to row 5 × 500 m intervals as fast as possible at resistance level 6 (Model D Indoor Rower, Concept2 Germany GmbH, Hamburg, Germany). Between the intervals, participants were given 1 min rest. After each 500 m interval, interval rowing time [sec], HR [bpm] (Polar H10, Polar Electro GmbH Deutschland, Büttelborn, Germany), and RPE were recorded. RPE was assessed with the Borg scale, which allows the expression of perceived exertion on a scale ranging from 6 to 20, whereby 6 represents the lowest and 20 the highest perceived exertion (Löllgen, 2004). HR was measured again 5 min after completion of the last interval. Blood lactate (Lactate Scout +, EKF Diagnostics, Barleben, Germany) was taken after completion of the rowing intervals (end lactate [mmol/L]) and again 5 min later (recovery lactate [mmol/L]). The total rowing time [sec] was calculated by summing up the rowing interval times of the 5 intervals. Based on HR and RPE measures of each interval, the average HR and RPE were calculated. HR drop [%] and lactate drop [%] were calculated to give an estimate for the recovery during the first 5 min after completion of the last interval. The study protocol is displayed in Figure 1.

The difference in total rowing time between the FBM and SBM conditions was the primary endpoint of this study. Secondary endpoints were differences in average HR and average RPE and in rowing interval times, RPE, and HR per interval between the FBM and SBM conditions. End lactate, recovery lactate, and the drop of HR and lactate during the first 5 min after completion of the rowing intervals were analyzed for exploratory reasons.

### 2.3 Participants

The study included healthy officers and officer candidates of the Federal German Armed Forces who were at least 18 years old studying sports science at the UniBw M. To achieve a power of 80% on a one-sided 5% significance level in the primary endpoint, a sample size of 22 was calculated by *a priori* power analysis with G*Power (Faul et al., 2007). In total, 26 participants signed up for the study, of which 5 participants were excluded (19%). Although 21 data sets were less than the calculated sample size needed, the post-hoc power analysis revealed that the power was still sufficient (0.81). After the pre-testing, participants were allocated to groups A (*N* = 10) and B (*N* = 11).

Of these 21 participants, 14% were female, and 86% were male. Their age ranged from 19 to 31 years. On average, the participants were 22.1 (SD = 2.7) years old, 180 (SD = 7.7) centimeters [cm] tall, weighed 82.1 (SD = 11.71) kilograms [kg], and did 3.3 (SD = 1.6) hours of endurance exercise per week. The study was conducted according to the guidelines of the Declaration of Helsinki and approved by the Ethics Committee of the UniBw M, Germany (06/04/2018). The guidelines of the European General Data Protection Regulation have been implemented. From all participants, consent was obtained before participation in the study.

### 2.4 Statistical analysis

Differences in total rowing time, average RPE, average HR, end lactate, HR recovery, and lactate recovery between the FBM and SBM conditions were analyzed with the Wilcoxon signed-rank test as the assumptions for normality were violated. Normal distribution was analyzed with Q-Q-plots and Shapiro–Wilk test. Within-group 5 × 2 repeated measures analyses of variances (rmANOVA) were conducted to analyze the change of rowing times, RPE, and HR per interval between the FBM and SBM condition (Blanca et al., 2017). The homogeneity of groups was tested via Levene’s test. Greenhouse–Geisser correction was applied if the assumption of sphericity was violated. Bonferroni correction was used for post-hoc tests to control for multiple testing. Statistical significance was set at *p* ≤ 0.05. Effect sizes of the rmANOVA are given in partial ⴄ (Greasley and Lamont, 2006). Data analysis was conducted with Excel 2019 (Microsoft, Redmond, United States) and JASP Version 0.16.4 (JASP, Amsterdam, Netherlands).

## 3 Results

### 3.1 Primary endpoint

The mean total rowing time when listening to SBM was 593 (SD = 74.3) sec. With a mean total rowing time of 572.9 (SD = 57.9) sec while listening to FBM, a significant difference in performance between music conditions was found (*p* = 0.009, r_B_ = 0.59). Additional information is presented in Table 1.

**Table 1:** Primary, secondary, and exploratory outcomes for the fast and slow beat music condition.

		Median	Mean	SD	Min	Max	*p*
Total time (sec)	SBM	583.00	593.05	74.33	484.00	772.00	0.009
	FBM	555.00	572.86	57.88	490.00	707.00	
Average HR (bpm)	SBM	177.20	173.31	14.30	125.00	195.20	0.03
	FBM	176.80	177.45	9.7	163.20	197.60	
Average RPE (score)	SBM	14.00	14.40	2.06	11.00	17.20	0.47
	FBM	14.60	14.50	1.55	12.20	16.80	
End lactate (mmol/L)	SBM	8.70	8.1	3.30	1.60	14.30	0.05
	FBM	10.00	9.21	3.01	3.80	13.90	
Recovery lactate (mmol/L)	SBM	8.50	7.79	2.99	1.60	11.90	0.01
	FBM	9.80	9.08	2.99	4.10	13.80	
HR drop (%)	SBM	41.62	40.21	6.76	19.16	49.46	0.05
	FBM	38.98	37.62	5.09	27.61	45.11	
Lactate drop (%)	SBM	2.3	1.91	12.59	−29.76	24.14	0.21
	FBM	−0.81	0.11	16.15	−30.19	28.06	

bpm, beats per minute; sec, seconds; mmol/L, millimoles per liter; FBM, fast beat music; SBM, slow beat music; HR, Heart Rate; SD, standard deviation; Min, Minimum; Max, Maximum; RPE, relative perceived exertion.

### 3.2 Secondary endpoints

**Figure 2:**
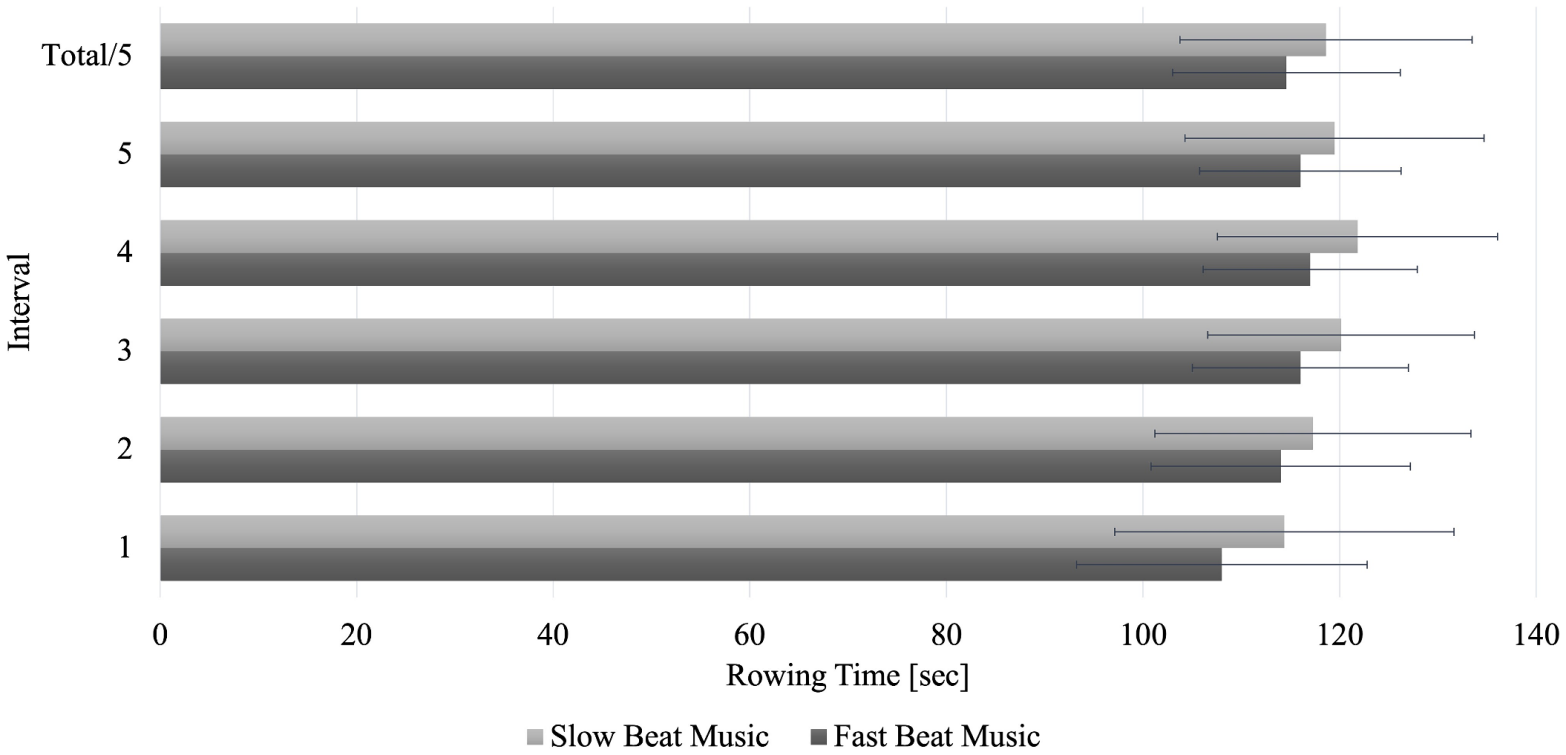
Rowing times for each interval split for the fast and slow beat music conditions. The upper bar “Total/5” shows the average rowing time over all 5 intervals for both conditions. sec, seconds.

Further analysis of rowing interval times via 5 × 2 rmANOVA with Greenhouse–Geisser correction revealed that there was no interaction effect (*p* = 0.45, η_p_^2^ = 0.04). Furthermore, no main effect for the factor *music* was found (*p* = 0.063, η_p_^2^ = 0.16). However, a significant main effect occurred for the factor *interval* (*p* < 0.001, η_p_^2^ = 0.49). Post-hoc tests with Bonferroni correction showed that the 1st interval was significantly faster than the intervals 2–5 (*p* < 0.001). The greatest difference in rowing times between the FBM and SBM condition was found for the 1st interval. The largest drop in performance occurred during the FBM condition between the 1st and 2nd interval. Total rowing times as well as times per interval are displayed in Figure 2.

**Figure 3:**
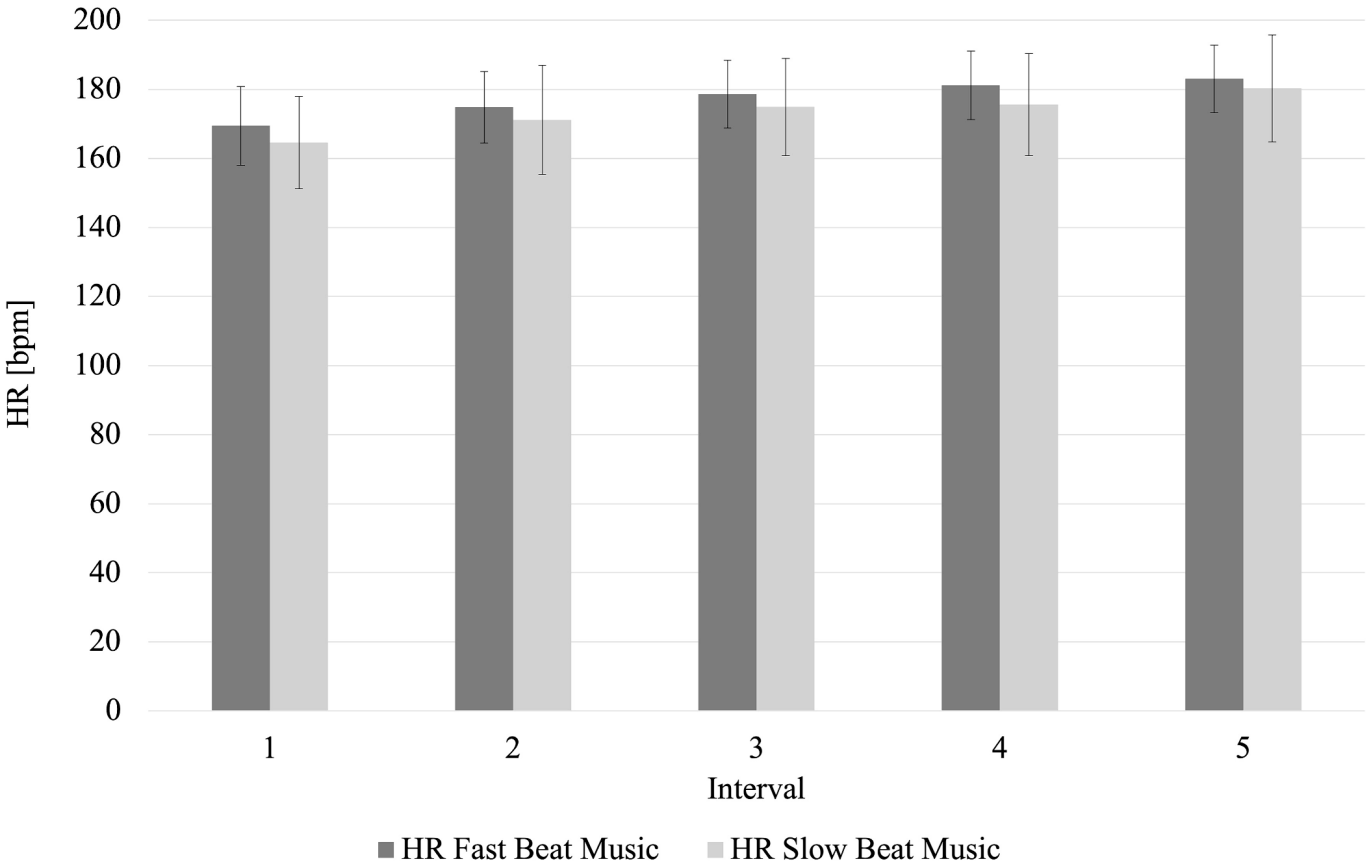
Relative perceived exertion over the course of the rowing intervals for the fast and slow beat music condition. HR Fast Beat Music, heart rate during fast beat music; HR Slow Beat Music, heart rate during slow beat music.

Mean average HR was 173.3 (SD = 14.3) bpm in the SBM condition and 177.5 (SD = 9.7) bpm in the FBM condition. This resulted in a significant difference in average HR between conditions (*p* = 0.03, r_B_ = 0.48). Looking at the 5 × 2 rmANOVA with Greenhouse–Geisser correction, there was no interaction between the factors interval and music (*p* = 0.44, η_p_^2^ = 0.04). Again, no significant main effect was found for the factor *music* (*p* = 0.10, η_p_^2^ = 0.13), but for the factor *interval* (*p* < 0.001, η_p_^2^ = 0.83). A post-hoc test with Bonferroni correction showed that in accordance with the rowing times per interval, the HR during the 1st interval was significantly higher than for the intervals 2–5 (*p* < 0.001). HR over the course of the 5 × 500 m rowing intervals are presented in Figure 3.

**Figure 4:**
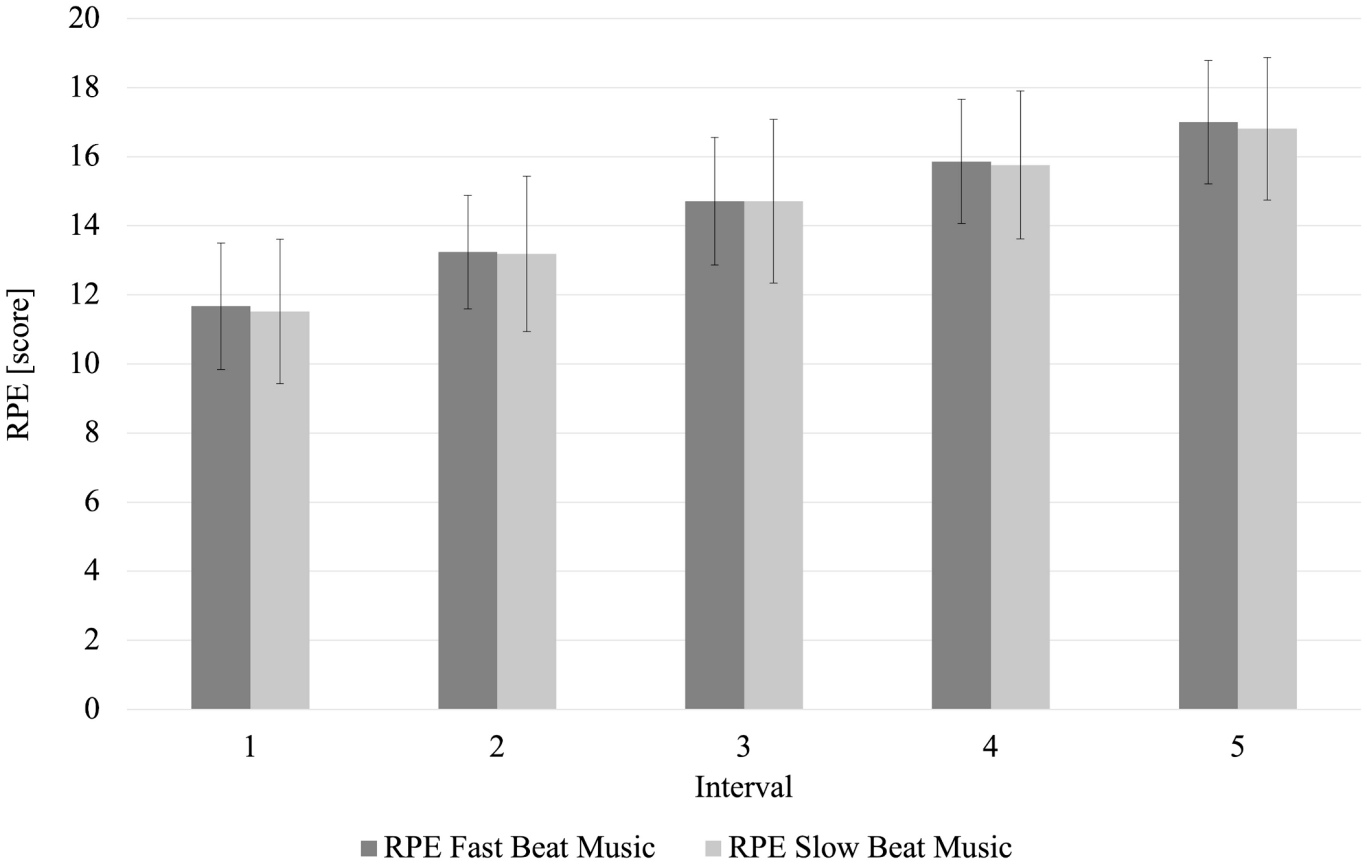
Heart rate and relative perceived exertion over the course of the rowing intervals for the fast and slow beat music condition. RPE Fast Beat Music, relative perceived exertion during fast beat music; RPE Slow Beat Music, relative perceived exertion during slow beat music.

In contrast to a significant higher average HR in the FBM condition, no significant difference in average RPE between groups was found (*p* = 0.47, r_B_ = 0.02). The interaction effect was also not significant (*p* = 0.97, η_p_^2^ = 0.01). A significant main effect occurred for the factor *interval* (*p* < 0.001, η_p_^2^ = 0.89), but not for *music* (*p* = 0.73, η_p_^2^ = 0.01). Post-hoc tests with Bonferroni correction showed that the RPE in the 1st interval was significantly lower than the RPE in the intervals 2–5 (*p* < 0.001). RPE over the course of the 5 × 500 m rowing intervals are presented in Figure 4.

### 3.3 Exploratory endpoints

The average lactate values after completion of interval 5 were 8.1 (SD = 3.3) mmol/L in the SBM and 9.2 (SD = 3) mmol/L in the FBM condition resulting in a significant difference between conditions (*p* = 0.05, r_B_ = 0.41). Lactate values 5 min after completion were 7.8 mmol/L (SD = 3) and 9.1 mmol/L (SD = 3) in the SBM and FBM condition. The blood lactate drop did not differ significantly between conditions (*p* = 0.21, r_B_ = 0.21). Contrary, the HR drop differed significantly between the SBM and FBM condition with a higher drop rate in the SBM condition (*p* = 0.05, r_B_ = 0.42). HR and lactate drop rates can be found in Table 1.

## 4 Discussion

The aim of the present study was to examine how exposition to SBM and FBM influences performance, physiological parameters, RPE, and recovery during high-intensity rowing intervals. In terms of performance, a positive effect of FBM was found compared to SBM. This was evident in each of the 5 intervals by means of shorter times to completion. This generally supports the notion of Karageorghis and Priest (2012a,b) that carefully selected music leads to beneficial ergogenic effects.

Indeed, comparisons with earlier studies should be treated with caution due to the small number of studies investigating high-intensity interval exercise and differing research designs (Marques et al., 2024). Nevertheless, some parallels can be drawn with a study of Rendi et al. (*N* = 22) who found that listening to FBM leads to greater performance during high-intensity rowing compared to SBM or no music. However, in the study of Rendi et al. participants performed only one 500 m sprint per music condition (FBM, SBM, no music) instead of multiple intervals (Rendi et al., 2008). Therefore, the results of the study conducted by Rendi et al. could at most be related to the results of the 1st interval of the present study. Interestingly, the difference in performance between FBM and SBM was most pronounced in the 1st interval. Apparently, the FBM could have acted as a more effective external psychological stimulus compared to the SBM that motivated the participants to be more willing to exercise hard.

In a qualitative study (*N* = 13) on the effects and characteristics of music accompanying exercise, Priest and Karageorghis showed that even sheer anticipation of a motivational music segment in the absence of music could have a stimulating effect (Priest and Karageorghis, 2008; Karageorghis and Priest, 2012a,b). In the current study, the participants knew whether the FBM or SBM would be played during the test. Therefore, they may have already been primed for high performance when expecting the motivational FBM. Because the influence of music is most evident when the physical task is self-paced, the effects observed in the present study could have been further amplified (Karageorghis and Priest, 2012a,b).

Under both music conditions, the 1st rowing interval was the fastest. Thereafter, times to completion increased until the 4th interval which indicates that the fatigue accumulated over time. Nevertheless, both groups were able to motivate themselves towards slightly shorter times to completion in the 5th compared to the 4th interval. Looking at completion times, it is worth noting that although the FBM was superior in terms of performance, especially in the 1st interval, participants also experienced the greatest drop in performance in the FBM condition, namely between the 1st and 2nd interval. Hutchinson et al. made a similar observation since they found that the energizing effects of music were strongest at the start of a physical task whereas this effect diminished over time (Hutchinson et al., 2011). Based on these findings, it is to assume that particularly FBM could increase the risk for over pacing which might impair the overall performance of athletes when performing multiple intervals or longer duration exercise.

Consequently, greater performance in the FBM condition was accompanied by higher mean HR and mean end lactate values, indicating that the participants were actually exerting themselves more physiologically compared to the SBM condition. However, RPE did not differ between conditions. This appears to contradict the theory that external sensory stimuli cannot be processed effectively during high-intensity exercise as the overwhelming physiological stimuli already overtax the capacity of the afferent nervous system (Rejeski, 1985; Hutchinson and Tenenbaum, 2007; Karageorghis and Priest, 2012a,b). Nevertheless, the FBM still seemed to distract participants from the higher physiological exertion which was also observed in previous research (Rendi et al., 2008). Following the theory of limited processing capacity, it could be assumed that rowing indoors on an ergometer is not that taxing for the afferent nervous system due to the low technical demands of the exercise as well as minimal external (e.g., changing scenery, people, odours) and internal (e.g., kinaesthetic) stimuli to be processed when compared to translational movements performed outdoors. Furthermore, while rowing 500 m at high intensities is indeed a demanding physical task, participants did not experience the same level of physiological fatigue over the course of an interval. Instead, physiological fatigue built up over the course of each interval and declined again between the intervals. As a result, for a certain period of time during each interval, physiological feedback signals might have been low enough for the participants to still profit from the ergogenic and psychophysical effects of the FBM. Additionally, the faster time to the completion in the 5th compared to the 4th interval further indicates that participants did not reach volitional fatigue, at least in the intervals 1–4. This must be taken into account in order to compare the results of the present study with those of previous investigations, in which the participants exercised at high intensities as well, but had to maintain a high intensity until volitional exhaustion (Eliakim et al., 2012; Maddigan et al., 2019).

In terms of recovery, results of the current study suggest that listening to SBM facilitates HR drop after high-intensity exercise. This could be either attributed to a reduction in physiological arousal by the SBM or the inhibition of FBM on HR recovery following high-intensity exercise (Karageorghis et al., 2018; de Witte et al., 2020; Terry et al., 2020).

In the present study, the strategies Marques et al. recommended in a recent review to benefit performance, RPE, and recovery during high-intensity exercise were applied (Marques et al., 2024). This included the application of self-selected, preferred motivational music that was played throughout the complete protocol consisting of several high-intensity bouts. The novelty of this study lies in the application of these strategies during high-intensity rowing ergometer intervals with solely the music tempo being varied. Contrastingly, earlier studies usually carried out cycle-based tests with shorter intervals (Stork et al., 2015; Maddigan et al., 2019; Stork et al., 2019; Marques et al., 2022). Studies using a rowing ergometer on the other hand did not set up high-intensity protocols with multiple bouts (Rendi et al., 2008; Karow et al., 2020). Accordingly, the research design applied in the present study expanded the current state of research. It was shown that FBM has a positive effect on performance without increasing RPE during high-intensity rowing ergometer tests compared to SBM, even if several intervals had to be completed. The results of the present study further suggested that the positive effects of FBM on performance and RPE can also be observed during self-paced high-intensity exercise when the interval lengths exceed the duration investigated in previous studies (Stork et al., 2015; Maddigan et al., 2019; Stork et al., 2019; Marques et al., 2022). As this study additionally examined the recovery phase after the last interval, it was possible to extend the knowledge regarding music tempo on recovery following high-intensity bouts.

Some limitations should be considered when interpreting the results of the current study. First, we did not include a no-music condition, which makes it difficult to estimate the effect of music in general on the parameters assessed. In addition, this would allow for more in-depth comparisons of the present study with previous ones. While few studies explicitly compared FBM with SBM, several studies investigated the effects of FBM versus no music during high-intensity exercise (Pujol and Langenfeld, 1999; Maddigan et al., 2019). Further, the participants were not homogeneous in terms of their level of training, which should be taken into account, as the benefit of music differs depending on training status (Brownley et al., 1995; Mohammadzadeh et al., 2008; Terry et al., 2020). Additionally, it should be noted that albeit the exercise chosen was rowing on the ergometer, the participants were not rowers but physically active sport science students. Therefore, the results can not necessarily be generalized to other groups of people. Lastly, blood lactate was not measured after each interval, although this would have provided valuable insight regarding the interpretation of physiological fatigue over time. According the findings of this study as well as the mentioned limitations, it is recommended to integrate a no-music condition in future studies. Furthermore, research should consider including physically inactive adults as well as recreational or professional rowers. It is further recommended to investigate different high-intensity protocols and measure lactate values after each interval.

In conclusion, FBM should be preferred during high-intensity rowing intervals to improve performance without an elevation in RPE. In contrast, SBM was superior in terms of recovery after exercise completion. Therefore, systematic use of music that is tailored to the intended training stimulus offers broad opportunities for competitive sports as well as the fitness and health sector. However, further research is required to estimate the effects of deliberate manipulation of music in the aforementioned exercise scenarios.

## 5 Practical applications

Several possible applications with practical relevance for sports and exercise can be derived from the results of the current study. Distinctions must be made between the conditions in which music could be used to induce certain ergogenic and psychophysical effects. Firstly, music could serve as a tool to help athletes performing high-intensity interval training as part of their training to reach the desired state that matches the current training intention. For example, FBM could be applied during high-intensity interval training whereas SBM might profit recovery periods or deload sessions. It is also conceivable that coaches manipulate music if they realize that their athletes are over pacing or underperforming during training sessions. In this way, verbal instructions could be supplemented by an additional subliminal stimulus in the form of FBM or SBM, without the athletes being aware of this. In competition, when athletes can not select their preferred music or when no music is played at all, SBM could still be used during pauses or post intense competition to aid recovery.

Besides competitive sports, there are further possible applications of music in fitness regimes which regularly include high-intensity interval training such as CrossFit^©^ or Hyrox^©^ (Glassman, 2020; Bergmann, 2024). FBM could help participants display greater performance during high-intensity workouts helping them to achieve their health and fitness goals. In CrossFit^©^ as well as Hyrox^©^, physiological demands are diverse and vary over the course of different training sessions or even within a single session. Therefore, well selected music could prove beneficial (Meier et al., 2022). In case of a 60 min CrossFit^©^ session, SBM could be played during the warm-up to avoid undesired fatigue. FBM could accompany the high-intensity part of the workout to improve performance and training stimuli while simultaneously curbing perceived exertion. During the cool-down, SBM may be used again to aid recovery. Similar music accompaniment would be imaginable for other fitness areas. Since especially untrained individuals are affected by music, such approaches could provide a considerable opportunity to improve the enjoyment of exercise in physically inactive populations to promote exercise participation and adherence (Brownley et al., 1995; Mohammadzadeh et al., 2008). It is recommended to apply the strategies suggested by Marques et al. Marques et al. (2024) if possible.

## Data availability statement

The data that support the findings of this study are available from the corresponding author upon reasonable request.

## Ethics statement

The studies involving humans were approved by Ethics Committee of the University of the Bundeswehr Munich, Germany (06/04/2018). The studies were conducted in accordance with the local legislation and institutional requirements. The participants provided their written informed consent to participate in this study.

## Author contributions

AndS: Data curation, Formal analysis, Visualization, Writing – original draft. TB: Data curation, Formal analysis, Visualization, Writing – original draft. DA: Investigation, Writing – review & editing. PA: Investigation, Writing – review & editing. MF: Methodology, Writing – review & editing. AnnS: Conceptualization, Methodology, Writing – review & editing.
